# Adverse events of repetitive transcranial magnetic stimulation in older adults with depression, a systematic review of the literature

**DOI:** 10.1002/gps.5440

**Published:** 2020-11-06

**Authors:** Geke M. Overvliet, Rebecca A. C. Jansen, Anton J. L. M. van Balkom, Dilene C. van Campen, Mardien L. Oudega, Ysbrand D. van der Werf, Eric van Exel, Odile A. van den Heuvel, Annemiek Dols

**Affiliations:** ^1^ Department of Old Age Psychiatry GGZ inGeest Specialized Mental Health Care Amsterdam Netherlands; ^2^ Department of Neurology Amsterdam UMC location VUmc Amsterdam Neuroscience Amsterdam Netherlands; ^3^ GGZ inGeest Specialized Mental Health Care Amsterdam Netherlands; ^4^ Department of Anatomy & Neurosciences Amsterdam UMC location VUmc Amsterdam Neuroscience Amsterdam Netherlands; ^5^ Department of Psychiatry Amsterdam UMC location VUmc Amsterdam Neuroscience Amsterdam Netherlands

**Keywords:** adverse events, late life depression, older adults, repetitive transcranial magnetic stimulation, serious adverse events

## Abstract

**Objective:**

In the last decade, repetitive transcranial magnetic stimulation (rTMS) has been introduced as a non‐invasive neuromodulation therapy for depression. Little is known, however, about (serious) adverse events (AE) of rTMS in older adults with a depression. In this article, we want to study what is known about (serious) AE of rTMS in older adults (>60 years) with late‐life depression (LLD).

**Methods:**

A systematic search has been performed according to the PRISMA guidelines in PubMed, EMBase and PsycInfo. We have screened 622 articles for eligibility. Eleven studies, evaluating 353 patients in total, were included in this review.

**Results:**

AE were reported in 12.4% of the older adults with a LLD treated with rTMS, serious AE in 1.5%. Headache (6.9%) and discomfort at the stimulation site (2.7%) are the most commonly reported AE. Serious AE reported are: psychiatric hospitalization (three times), a combination of posterior vitreous detachment and retinal tear, and increased suicide ideation (both once).

**Conclusions:**

rTMS in older adults with LLD was concluded overall to be safe due to the low frequency of AE reported in trials and observational studies. In case‐reports, however, more serious AE have been described. To tailor use of rTMS in older adults with LLD, more research is needed in larger samples to optimize tolerance.

## Highlights

- Little is known about (serious) AE of rTMS in older adults (>60 years) with LLD.
- A relatively low percentage of AE (12.4% in total) and serious AE (1.5% in total) in response to rTMS occurs in older adults with LLD.
- rTMS is a safe and well‐tolerated treatment option for older adults with LLD.

**Abbreviations**
AE: adverse events
DLPFC: dorsolateral prefrontal cortex
FDA: Food and Drug Administration; HF, high frequency; LF, low frequency
LLD: late‐life depression; NICE, National Institute for Health Care Excellence
RCT: randomized controlled trial
rTMS: repetitive transcranial magnetic stimulation; VLPFC, ventrolateral prefrontal cortex

## 1 INTRODUCTION

Today's society is aging rapidly. In the upcoming 30 years, the proportion of the world's population aged above 60 years will probably rise from 12% to 22%.^1^ The number of older adults who suffer from mental illness is rising as well, based on long‐term cohort studies that report 22%.^2^ One of the most common mental disorders in this age group is depression, next to dementia. Depression most often causes substantial suffering and a decrease in quality of life. Late‐life depression (LLD) is a primary diagnosis of major depression, dysthymia or minor depression according to DSM‐IV criteria, in patients aged 60 years and over.^3^ Especially in older adults, treatment of LLD is challenging as vulnerability for adverse events (AE) of anti‐depressive medication increases with age.^3^ Treatment options for this vulnerable group should be carefully assessed. A rapidly increasing new treatment option for depression is repetitive transcranial magnetic stimulation (rTMS), a noninvasive brain stimulation technique.

With rTMS, a rapidly changing magnetic field is used to generate an electric current in the brain tissue just below the skull, to alter the cortical excitation of this brain region and its interconnected brain network.^4^ rTMS was approved by the Food and Drug Administration for treatment of mild to moderate treatment‐resistant depression in 2008, but the first TMS device has been developed already during the early 1980s.^5^

To date, rTMS is not a standard treatment option in the general population and only recommended by some guidelines with caution,^6^ as for example in the Dutch multidisciplinary depression guideline.^7^, ^8^ A Cochrane review published in 2002 mentioned that there was no strong evidence for benefit from using rTMS to treat depression, although the small sample sizes did not exclude the possibility of benefit.^9^ There are no open consensus guidelines available from the American Psychiatry Association.^10^ The clinical TMS society stated some treatment recommendations based on a literature of three RCT's and a user survey of 68 members of the clinical TMS society. They suggest that TMS therapy is recommended and should be considered as an acute treatment for symptomatic relief of depression in patients who have failed to receive satisfactory improvement from prior antidepressant medication in the current episode.^11^ For the best results of an rTMS protocol, knowledge of the pathology is important. For example, there is an imbalance between the left and right hemisphere in depression; the left dorsolateral prefrontal cortex (DLPFC) is known to be hypoactive.^12^ The application of high‐frequency left rTMS (HF left rTMS) or low frequency right rTMS (LF right rTMS) both have anti‐depressive effects. Different stimulation frequencies are thought to exert their effects through a differential influence, that is, increasing or decreasing excitability.^12^

The use of rTMS as a treatment is increasing, also in older adults. Nevertheless, extensive clinical research in the older adult population is sparse, especially when it comes to (serious) AE.^6^ Higher prevalence of AE in the older population is expected as this patient group suffers far more than younger adults from physical comorbidity and polypharmacy.^13^ Comorbidity and polypharmacy are common exclusion criteria in clinical trials, but frequently present in clinical practice with older adults. The exclusion of comorbidity and polypharmacy causes a possible underestimation of prevalence of (serious) AE in clinical trials.

Research on the efficacy of rTMS has shown response rates of 20% to 50% in older adults with LLD,^14^ similar to response rates in adults with treatment‐resistant depression.^15^ Studies on the tolerability of rTMS in adults has reported several AE such as headache (9.7%), local pain and discomfort (9.3%), and neck pain, toothache, and paresthesia (together 4.7%).^16^ Rare serious AE (<1%) are seizures and induction of hypomania, hearing changes and burns from the coil.^4^, ^14^, ^17^, ^18^, ^19^, ^20^, ^21^

Most often, people aged over 60 are excluded from trials. The National Institute for Health Care Excellence (NICE)‐guideline of rTMS for depression is based on studies with a mean age between 38.4 and 50.5 years, the remaining studies used in the NICE‐guideline did not mention a mean age.^6^ To date, several publications exist about efficacy of rTMS in older adults with LLD, but no studies have systematically examined tolerability and safety of rTMS for LLD.^14^ The rationale for this review is to give an overview about the (serious) AE of rTMS in LLD. In this review, we consider reports of patients that are older adults with a LLD, use rTMS as intervention, use sham rTMS (when available) as comparison and report (serious) AE as outcome.

## 2 MATERIAL AND METHODS

The study was performed according to the PRISMA‐guidelines.

### 2.1 Eligibility criteria

Study characteristics: We included studies that investigated patients who were older adults with LLD, used rTMS as intervention, used a comparison consisting of (when available) sham rTMS with real rTMS, in which (serious) AE was reported as an outcome or had a study design that is a review of the existing literature on this topic to check the references of that review. Studies that included patients with brain damage, such as tumors and brain contusions, were excluded. We included articles of the last 15 years. We excluded meta‐analyses, and articles written in another language than English as well.

A search was executed on January 16th 2019 in PubMed, PsycInfo/Ebsco, and EMBase on the and updated on 23th of November 2019. Mesh Terms and free text terms [tiab] were used. Also, the option ‘Similar articles’ in PubMed was used. For the search we used the following keywords: adverse effects, side effects, harmful effects, AEs, safety, headache*, nightmare*, somnolence*, pain, mania, convulsion, transcranial magnetic stimulation, repetitive transcranial magnetic stimulation, rTMS, aged, elderly, older adult*, elder and geriatric*. For the study selection we removed the duplicates first. Second, we screened the records on title and abstract. Then we read full‐text articles assessing for eligibility. Finally, we performed a snowball search to select articles, by checking the references of the articles used for this review, that may have been missed in the primary search.

### 2.2 Data collection process

Two authors performed data extraction (RJ & GO) independently, that was subsequently compared when different results were found. The following data items were acquired: year of publication, number of patients, age of patients, stimulation parameters used during rTMS (type of rTMS, location of rTMS, frequency and duration of rTMS, and rTMS details), and (serious) AE. We included all (serious) AE as reported as such in the publications found by the search as the principal summary measure.

### 2.3 Synthesis of results

To combine the results of the studies, we counted all the reported (serious) AE and divided the number by that of all the included patients.

### 2.4 Risk of bias of individual studies

We assessed the risk of bias of the included studies by using the recommended Cochrane Collaboration's Risk of Bias evaluation tool, that evaluates the bias in the conducted studies on reporting results, in this case: AEs. Using this tool, two independent authors in a double blind fashion (RJ & GO) scored six types of bias (selection bias, performance bias, detection bias, attrition bias, reporting bias and other types of bias) as low, high or unclear on potential risk of bias.^22^

## 3 RESULTS

### 3.1 Study selection

**FIGURE 1:**
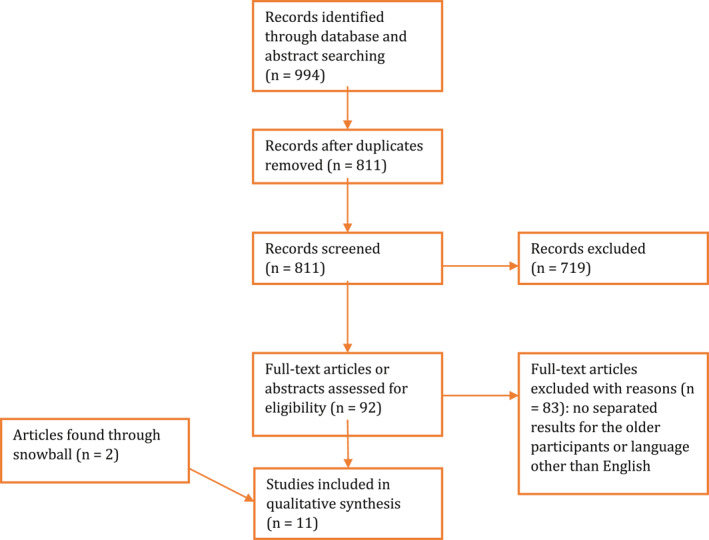
Flow diagram

A total of 994 Articles was found. After removing duplicates, 811 articles remained. We screened these articles for eligibility by reading the title and abstract, resulting in 92 articles. These articles were full‐text screened, eight articles were identified as eligible. Through snowball search two additional articles and two case‐reports were found. We excluded one study,^23^ because the study population appeared to be a subsample of another included study.^24^ In total, 11 studies were included. There were two RCT's, four open label studies, two retrospective studies, two case series, and two case‐reports (Figure 1).

A total of 331 included older adults received rTMS and 49 older adults received a form of sham treatment. Most older adults (*n* = 246) received high frequency rTMS (HF‐rTMS), 70 older adults received low frequency rTMS (LF‐rTMS), and 15 older adults received both HF‐rTMS and LF‐rTMS. None of the studies mentioned the use of theta burst stimulation in their methods.^25^ Sham condition was used in three studies, and performed by placing the coil at a 90° angle with the scalp, or the intensity setting was put on 0,0, or was not described. Specifying AE between stimulation frequencies was difficult due to the low number of older adults who received LF‐rTMS and the lack of specificity of reported AE in some studies. We therefore lumped the findings of serious AE of HF‐rTMS and LF‐rTMS together. Some studies reported the use of H1‐coils^26^; although an H‐coil can effectively stimulate deeper targets, it might activate different regions compared to standard figure‐of‐eight coils.^12^

### 3.2 Results of individual studies

#### 3.2.1 Randomized controlled trials

Trevizol and colleagues^27^ included 43 older adults with LLD in a randomized controlled trial (RCT). A total of 11 older adults with a mean age of 66.1 years (SD 8.5) received unilateral HF‐rTMS at the left DLPFC (10 Hz) and another 20 older adults with a mean age of 66.8 years (SD 5.8) received combined LF‐rTMS at the right DLPFC (1 Hz) followed by HF‐rTMS at the left DLPFC. The sham‐group included 12 older adults with a mean age of 64.1 years (SD 3.7). Sham was performed by placing the coil at 90 degrees of the scalp in a single wing tilt position, out of the view of the participants, creating an experience comparable with active rTMS. AE were only reported in the group treated with unilateral HF‐rTMS; one patient reported headache and one patient reported insomnia. One patient dropped out due to intolerance to the treatment and three patients dropped out due to lack of treatment response (see Table 1).

**TABLE 1:** Summary of the articles

Reference	Study population	Type rTMS and location	rTMS details	Treatment sessions	Side‐effects
Trevizol et al.^27^ 2019 RCT	20 older adults bilateral rTMS 66.8 years (SD 5.8). 11 older adults unilateral rTMS 66.1 years (SD 8.5) and 12 older adults sham treatment 64.1 years (SD 3.7)	HF‐rTMS or HF‐rTMS followed LF‐rTMS with B‐65 figure‐8 coil Location: HF‐rTMS left DLPFC and LF‐rTMS right DLPFC	Unilateral: Intensity: 120% of motor threshold, 1450–2100 pulses (48–70 trains) of 10 Hz Bilateral: Intensity: 120% of motor threshold, 750–1500 pulses (25–50 trains) of 10 Hz followed by 465–600 pulses (5–6 trains) of 1 Hz	15 (5 days a week, 3 weeks). Non‐remitters 30 (5 days a week, 6 weeks)	SAE: none AE: headache (1x), insomnia (1x) Dropouts: 4, (1 due intolerance of treatment, 3 due lack of response)
Kaster et al.^26^ 2018 RCT	25 older adults rTMS 65.0 years (SD 5.5),27 older adults sham treatment rTMS, 65.4 years (SD 5.5)	HF‐rTMS with H1 coil Location: DLPFC and VLPFC bilateral	Intensity: 120% of motor threshold, 6012 pulses (2s pulse train and 20s inter‐train interval, 167 trains) of 18 Hz versus sham rTMS	20 (5 days a week, 4 weeks)	SAE: none AE: headache after treatment (14x), pain at stimulation site/discomfort (4x), nasopharyngitis (1x), aphthous ulcer (1x), corneal abrasion (1x), dermatitis (1x)s, sinusitis (1x), nausea(1x) Dropouts: 5
Leblhuber et al.^28^ 2018 Sham controlled pilot study	19 older adults bilateral rTMS 71.9 years (SD 2.9), 10 older adults sham treatment 73.3 years (SD 2.7)	Not mentioned	Intensity, pulses and trains: Not mentioned. 3 Hz	10 (5 days a week, 2 weeks)	SAE: none AE: none Dropouts: none
Dardenne et al.^21^ 2018 Open pilot study	10 older adults, female 73.9 years (SD 5.7)	HF‐rTMS 70 mm figure‐8 coil. Location: Left DLPFC	Intensity: 110% of resting motor threshold, 1560 pulses (2s pulse train, 12s inter‐train interval) of 20 Hz.	20 (5 times a day, 4 days)	SAE: none AE: discomfort (1x) and headaches (4x) Dropouts: none
Sayar et al.^29^ 2013 Prospective trial	65 older adults 66.6 years (SD 5.77)	HF‐rTMS with figure‐8 coil. Location: Left prefrontal cortex	Intensity: 100% of motor threshold, 1000 pulses (20 trains of 2s pulse train, 30s inter‐train interval, of 25 Hz.	18 (6 days a week, 3 weeks)	SAE: none AE: none Dropouts: none
Pallanti et al.^30^ 2012 Prospective trial	36 older adults 67.2 years (SD 4.2). 66 younger patients 43.3 years (SD 9.8)	LF‐rTMS with 70 mm figure‐8 coil. Location: Right DLPFC	Intensity: 110% of rest motor threshold, 420 pulses (3 × 140s trains, 30s inter‐train interval) of 1 Hz	15 (5 times a week, 3 weeks)	SAE or AE: none* Dropouts: 19 × increased anxiety, insomnia, induced mood elevation and discomfort due to stimulation* *in the whole study population, not specified for age
Desbeaumes et al.^31^ 2018 Retrospective study	19 older adults 71.0 years (SD 8.3)	HF‐rTMS with Cool‐B65 coil.Location: Left DLPFC	Intensity: 110% of motor threshold, 3000 pulses (30 trains of 100 pulsations, 5s pulse train, 25s inter‐train interval) of 20 Hz	20–30 (2 times a day, 3–5 times a week)	SAE: none AE: headaches (3x), local sensitivity(3x) and fatigue (1x). Dropouts: 2
Conelea et al.^32^ 2017 Retrospective study	75 older adults 66.0 years (SD 5.5)	HF‐rTMS. Location: Left DLPFC	Intensity: 120% of motor threshold, 3000 pulses of 5 or 10 Hz	30–50 (5 times a week)	SAE: psychiatric hospitalization(3x), hospitalization unrelated to rTMS (1x) AE: none Dropouts: 3
Milev et al.^24^ 2009 Case‐series	49 older adults 69.4 years (SD 7.8)	HF‐rTMS, LF‐rTMS, HF + LF‐rTMS with figure‐8 coil or round coil Location, left DLPFC, right DLPFC or both	Intensity: 80%–110% of motor threshold, 1600 pulses (20 trains of 8s pulse train, 52s inter‐train interval of 10 Hz	10 (minimum of 8 sessions. Six patients received 14 sessions or more)	SAE: none AE: headache (1x) and discomfort (1x), slight transient discomfort (several) Dropouts: 1
Elmaadawi et al.^33^ 2016 Case‐report	One 60‐year‐old female	HF‐rTMS Location: Left DLPFC	Intensity: 3000 pulses in 36 min	29 (5 times a week)	SAE: increased suicide ideation AE: none Dropouts: ‐
Kung et al.^34^ 2017 Case‐report	One 65‐year‐old female	Low‐frequency rTMS with figure‐8 coil. Location: Right DLPFC	Intensity: 110% of motor threshold, 3000 pulses (three trains of 1000s, 60s inter‐train interval) of 1 Hz.	15	SAE: Posterior vitreous detachment and retinal tear AE: discomfort and eye twitching Dropouts: ‐

Abbreviations: AE, adverse events; DLPFC, dorsolateral prefrontal cortex; HF‐rTMS, high‐frequency repetitive transcranial magnetic stimulation; LF‐rTMS, low‐frequency repetitive transcranial magnetic stimulation; SAE, serious adverse events; VLPFC, ventrolateral prefrontal cortex.

Kaster and colleagues^26^ included 52 older adults aged 60–80 years with LLD in a RCT. A total of 25 older adults with a mean age of 65.0 years (SD 5.5) received active HF‐rTMS (18 Hz). In addition, 27 older adults with a mean age of 65.4 years (SD 5.5) received sham rTMS. The sham procedure was not described. HF‐rTMS sessions were delivered with the H1 coil targeting the dorsolateral and ventrolateral prefrontal cortex bilaterally. During treatment the following AE were reported in the active HF‐rTMS group: headache after treatment (n = 14, 56.0%), pain at the stimulation site (n = 4, 16.0%) nasopharyngitis, aphthous ulcer, corneal abrasion, dermatitis, sinusitis, and nausea (all n = 1, 4.0%). Pain at the stimulation site was the only AE significantly more present in the active HF‐rTMS group than in the sham rTMS group (16.0% vs. 0.0%). Five older adults in the active HF‐rTMS group dropped out: one patient dropped out due to discomfort from the stimulation, one patient dropped out because of a worsening of the depressive symptoms, three patients dropped out due to circumstances not related to HF‐rTMS.

#### 3.2.2 Open label studies

Leblhuber and colleagues^28^ performed a study with 19 older adults with LLD and a mean age of 71.9 years (SD 2.9) with bilateral HF‐rTMS at the DLPFC (3 Hz) and a sham group of 10 older adults with LLD and a mean age of 73.3 years (SD 2.7). The sham condition was not described. No AE were reported.

In an open‐labeled pilot study, Dardenne and colleagues^21^ included 10 older females with a mean age of 73.9 years (SD 5.7) diagnosed with LLD who failed to respond to at least one course of antidepressant treatment. The older females were treated with HF‐rTMS at the left DLPFC (20 Hz) without serious AE during the study. Reported AE were headache in four older adults and local scalp discomfort in one older adult.

Sayar and colleagues^29^ included 65 older adults with a mean age of 66.6 years (SD 5.8), with LLD who were treated with HF‐rTMS at the left prefrontal cortex (20 Hz) in a prospective trial. No AE nor dropouts were reported.

Pallanti and colleagues^30^ performed a study in 102 treatment‐resistant patients with depression, of which 36 older adults with a mean age of 67.2 years (SD 4.2). LLD was treated with LF‐rTMS at the right DLPFC (1 Hz). No AE were reported in those 36 older adults. There were some dropouts (due to anxiety, insomnia, induced mood elevation, increasing discomfort from the stimulation of the scalp, and the need for hospitalization during the protocol period); no details such as the exact number of these dropouts were available.

#### 3.2.3 Retrospective studies

Desbeaumes and colleagues^31^ included, in their retrospective study, 19 older adults with LLD with a mean age of 71.0 years (SD 8.3) treated with HF‐rTMS at left DLPFC (20 Hz). Five older adults (26.3%) reported AE such as headaches, local sensitivity and fatigue. Two of these older adults dropped out, although it was not mentioned why. One patient stopped because of local scalp sensitivity and another patient stopped because of a tremor, present already prior to treatment.

Conelea and colleagues^32^ included 75 older adults with a mean aged of 66.0 years (SD 5.5), diagnosed with treatment‐resistant LLD. The older adults were treated with HF‐rTMS at the left DLPFC (5 or 10 Hz). AE were retrospectively identified. Out of 75 older adults, three were admitted to a psychiatric hospital, although it was not mentioned if this was due to the psychiatric disease or due to the AE as a result of rTMS. One older adult was hospitalized unrelated due to the HF‐rTMS.

#### 3.2.4 Case series and case reports

In the study of Milev and colleagues,^24^ 49 older adults with LLD with a mean age of 69.4 years (SD 7.8), were treated with rTMS with varying combinations of HF‐rTMS at the left DLPFC (10 Hz, *n* = 31), LF‐rTMS at the right DLPFC (1 Hz, [*n* = 14] or a combination of both [*n* = 4]). No serious AE were reported, headache and discomfort were both reported once as an AE. One older adult dropped out.

Elmaadawi and colleagues^33^ reported a serious AE in a 60‐year‐old female with treatment‐resistant depression for more than 30 years, who was treated with HF‐rTMS at the left DLPFC. Prior to the HF‐rTMS, she did not have suicidal ideation or a plan. Her last suicidal ideation was a year before treatment started. She had never had a suicide attempt or previous psychiatric hospitalization. In the first two weeks of treatment, her depression and quality of life improved, but in the third week she reported suicidal ideations. The suicidal ideations became severe with an active plan. After 29 sessions HF‐rTMS was terminated and she was hospitalized to stabilize.

A possible serious AE during LF‐rTMS treatment was reported by Kung and colleagues^34^ in a 65‐year‐old Caucasian woman diagnosed with LLD. She was treated with LF‐rTMS at the right DLPFC (1 Hz). During the first ten sessions, she experienced two AE; right eye twitching and discomfort. It was not described what the exact location of the discomfort was. A few hours after the 11th treatment she experienced headache. At this moment there were no ophthalmologic symptoms at examination. Before the 12th session started a posterior vitreous detachment and a retinal tear were found in her right eye. After treatment of the posterior vitreous detachment and retinal tear the patient continued LF‐rTMS sessions. At the 15th session she experienced a shower of several black dots in her right eye for about 5 s. LF‐rTMS treatment was stopped after this experience. In this case the development of a posterior vitreous detachment and retinal tear is described after LF‐rTMS, but it is not clear if the LF‐rTMS was the cause of these serious AE.

### 3.3 Synthesis of results

To summarize, AE were reported in 41 (12.4%) of the 331 older adults included in this review. The most frequent AE was headache (*n* = 23, 6.9%), followed by pain at stimulation site (*n* = 10, 3.0%). Insomnia, nasopharyngitis, aphthous ulcer, corneal abrasion, dermatitis, sinusitis, nausea and fatigue were all mentioned once (0.3%). Serious AE, however, were reported in 5 of the 331 cases (1.5%)^32^, ^33^, ^34^; posterior vitreous detachment and retinal tear (once), increased suicidal ideation (once), and psychiatric hospitalization (three times, 0.9%). Most of the (serious) AE were in the HF‐rTMS group, except of the one (serious) AE of the study of Kung and colleagues,^34^ that was the only (serious) AE in the LF‐rTMS group.

### 3.4 Risk of bias assessment (Table 2)

We did not only assess the studies on content, but also on quality. By using the recommended Cochrane guidance,^22^ we found that most studies had a high risk of bias by selection bias based on their type of study, like open label studies, retrospective studies, case series, and case reports. Some studies are small or did not have a sham or control group. These biases have an impact on the generalizability of the findings, not only on the primary outcome measurement of those studies, but also on the secondary outcome measurements, in which (serious) AE are frequently described.

**TABLE 2:** Risk of bias

	Selection bias	Performance bias	Detection bias	Attrition bias	Reporting bias	Other bias
Trevizol et al.^27^ 2019	Low risk of bias	High risk of bias	Low risk of bias	Low risk of bias	Low risk of bias	Small sample size, unbalanced number in groups
Kaster et al.^26^ 2018	Low risk of bias	Low risk of bias	Low risk of bias	Low risk of bias	Low risk of bias	Small sample size, recruitment stopped before target sample size was reached, short follow‐up time,
Leblhuber et al.^28^ 2019	High risk of bias	High risk of bias	High risk of bias	High risk of bias	High risk of bias	AE and dropouts not structural reported in protocol or results, only in abstract
Dardenne et al.^21^ 2018	High risk of bias	High risk of bias	High risk of bias	Low risk of bias	Low risk of bias	Case series, small study population, no sham/control group, short follow‐up
Sayar et al.^29^ 2013	High risk of bias	High risk of bias	High risk of bias	Potential risk of bias	Low risk of bias	Prospective study, patients were prospectively informed about side‐effects, no sham/control group, short follow‐up
Pallanti et al.^30^ 2012	High risk of bias	High risk of bias	High risk of bias	High risk of bias	Low risk of bias	Small sample size, no sham/control group, short follow‐up
Desbeaumes et al.^31^ 2018	High risk of bias	High risk of bias	High risk of bias	Low risk of bias	Low risk of bias	Retrospective study, small sample size, no sham/control group, short follow‐up
Conelea et al.^32^ 2017	High risk of bias	High risk of bias	High risk of bias	Potential risk of bias	Low risk of bias	All outcomes reported, adverse events were retrospectively identified and not systematically captured
Milev et al.^24^ 2009	High risk of bias	High risk of bias	High risk of bias	Potential risk of bias	Potential risk of bias	No sham/control group, short follow‐up
Elmaadawi et al.^33^ 2016	High risk of bias	High risk of bias	High risk of bias	Low risk of bias	Low risk of bias	Case report
Kung et al.^34^ 2017	High risk of bias	High risk of bias	High risk of bias	Low risk of bias	Low risk of bias	Case report

Abbreviation: AE, adverse events.

## 4 DISCUSSION

### 4.1 Summary of evidence

In this article, we provide a systematic review of the current literature regarding the (serious) AE of rTMS in older adults (>60 years) who suffer from LLD. The main finding is that the majority of articles included in this review concluded that rTMS was a safe and well‐tolerated treatment option with only mild AE for LLD after initial screening. Some studies and case reports reported serious AE like suicide ideations, posterior vitreous detachment and a retinal tear. AE, consisting mainly of headache and pain at the stimulation side were reported in 41 (12.4%) of the 331 older adults included in this review. It is Important to note that HF‐rTMS and LF‐rTMS are two different methods in terms of action mechanism. Although the underlying mechanism is different, they are both used for the same clinical effect; improvement of LLD. Because the numbers of included studies and included patients are low, we lumped the results of both techniques together. However, given their different mode of action their comparability is limited. Based on the results here most of the (serious) AE were in the HF‐rTMS group (18.7% of the HF‐rTMS population), except the one (serious) AE from the study of Kung and colleagues^34^ in the LF‐rTMS group (1.4% of the LF‐rTMS population).

Serious AE were reported in 5 of the 331 cases (1.5%)^32^, ^33^, ^34^; posterior vitreous detachment and retinal tear (one older adult), increased suicidal ideation (one older adult), and psychiatric hospitalization (three older adults). It is still unclear, however, whether a causal relationship between rTMS and some of these serious AE exists, like the posterior vitreous detachment and retinal tear. There may be an indirect effect through increased intra ocular pressure, caused by rTMS evoking these ophthalmological problems.^34^ With respect to mortality as the most serious AE event that can occur, no deaths have been reported as (serious) AEs in any of the studies. This makes the mortality rate zero in older adults with LLD receiving rTMS.

The number of serious AE is low if we compare it with the absolute risk for all‐cause mortality over 1 year in older adults with a depression not taking antidepressants (7.0%), for those taking tricyclic antidepressants (8.1%), for those taking selective serotonin reuptake inhibitors (10.6%), and for taking other antidepressants (11.4%).^13^ Mortality rates in rTMS are not described or structurally studied until now. It could be that the mortality rates in rTMS are so low that it is missed in the current studies. We did not find anything about mortality rates in rTMS in the literature while we performed the study.

In general practice, older adults do not meet the exact criteria of a research protocol, as they have more comorbidity, polypharmacy, and long‐term treatment. Older adults use more medication, have more somatic comorbidity, and experience more frailty.^3^ It is reasonable that an interaction of these factors can cause AE, although scientific evidence is lacking. Until now, there was no structural overview of (serious) AE in older adults with depression receiving rTMS. In some studies, AE were described as a second outcome measurement. In case reports (serious) AE were described as the main topic of the case report.^33^, ^34^ The incompatibility between on the one hand the results of the trials and on the other hand the results of the case reports is remarkable. There are several explanations. First, all clinical trials have extensive exclusion criteria, thereby including only relatively healthy participants. The case reports contain cases that are probably more representative of daily practice. Second, the trials include relatively small sample sizes. Although the numbers of case reports are even smaller, one can wonder if the number of included patients are large enough to reach enough power. Third, not all AE of rTMS in the studies might have been measured or reported, as study protocols do not focus on AE, but on the effectiveness of an intervention. Some study protocols mentioned dropouts, but did not mention the reason for dropouts (except of the study of Trevizol and colleagues^27^). In total, there percentage of registered dropouts was 3.9%. These dropouts were not included them in their follow‐up. These patients could be dropouts due to (serious) AE. On the other hand, one might expect that patients who drop out of studies due to (serious) AE are usually reported as such in the study or referenced to an appendix of an institutional review board. Fourthly, the number of sessions given in most studies is not representative for the number given in daily practice. The protocol of most included studies comprised 10–20 treatment sessions versus 29 sessions in the case report of Elmaadawi et al.^33^ A lot of AE appear early in the treatment phase of rTMS, but some AE may appear later on in the treatment phase.

Finally, stimulation intensity, location of stimulation, position of stimulation and number of sessions and stimuli potentially influence the occurrence of (serious) AE. In addition, patients specific factors like age, medication use, and brain morphology (e.g., functional connectivity and neurodegeneration) will also influence the occurrence of (serious) AE.^35^ Consistent reports on AE are needed to describe such potential relationships. In addition, further research is warranted to find predictors for (serious) AE in rTMS.

To obtain more insight on the timeframe of development of AE, more longitudinal studies are needed, with systematic reporting of AE and their timing.

The comparison between the meta‐analysis on rTMS and (serious) AE in adults with our findings on older adults clearly showed the under‐representation of older adults in this type of research. Slotema and colleagues^16^ included in a meta‐analysis 40 studies of rTMS in adults with depression, but another 120 studies were excluded for several reasons. In total there were 1042 adults, divided in subgroups of 472 adults receiving HF‐rTMS at the DLPFC, 109 adults receiving LF‐rTMS at the DLPFC, and 461 adults receiving sham treatment. With larger sample sizes this type of analyse gains in strength. In a study population with 472 adults receiving HF‐rTMS at the DLPFC, 9.7% reported headache, 9.3% experienced scalp discomfort, 1.9% facial twitching, 1.5% tears in their eyes, 1.3% local erythema, 2.5% drowsiness, and 4.7% reported other AE. These percentages were lower in the study population of 109 adults receiving LF‐rTMS at the DLPFC of the meta‐analysis of Slotema and colleagues.^16^

If we compare the results of the adult population of Slotema and colleagues^16^ with the population described in this overview of 331 older adults receiving rTMS with the HF‐rTMS at the DLPFC, we found lower percentages; 6.9% experienced headache, 2.7% local or scalp discomfort, 0.3% fatigue or drowsiness, and 3.9% experienced other AE or serious AE. These percentages are lower in older adults and can be explained by several hypotheses. It could be that the number of studies is too small, AE are not well registered by number, or the rTMS protocols used are heterogeneous. On the other hand, it can be that the ageing process protects the older adults from AE. Another possible cause is that older adults report fewer AE, because they are used to it, regard rTMS as a last treatment option for help, and accept AE better.^36^ A systematic comparison between adults and older adults has never been done and it is hard to determine results on this matter.

Slotema and colleagues^16^ analysed in their meta‐analysis also the number of dropouts in the treatment of rTMS for depression. They found a percentage of dropout of 10.6% in the HF‐rTMS DLPFC group and of 8.3% in the LF‐rTMS DLPFC group. Based on the studies done in older adults, the dropout percentage of 3.9% seems to be lower than in the adult population, although research to compare these results has never been performed.

### 4.2 Limitations

Our intention was to compare different subgroups of older adults with a depression receiving rTMS and take into account several possible contributing factors that cause (serious) AE. It proved difficult to compare the results of the separate studies due to the diversity of the stimulation protocols: high‐frequency, low‐frequency, different stimulation sites and different intensities of motor threshold, because of insufficient samples and incomplete reports in previous literature. By using the Cochrane guidance for the Risk of bias evaluation^22^ we found that most of the studies had a high risk of bias. The selected studies included only two RCTs.^25^, ^27^ Only three studies^26^, ^27^, ^28^ did have a sham treatment group. Almost all included studies had a small sample size, were case series or case reports. The small reported effect sizes and limited power of the studies hamper the outcome of the current review. AE might be underreported in the types of clinical trials that have been used in this review. This may affect the results.

## 5 CONCLUSION

In conclusion, rTMS is a safe and well‐tolerated treatment option for older adults with LLD with a relatively low percentage of AE (12.4% in total) and serious AE (1.5%), based on the findings in this review. The most commonly reported AE are headaches, and pain at the stimulation site. Serious AE found in trials and case reports are psychiatric hospitalization, suicide ideation, retinal tear and posterior vitreous detachment. Routine assessment and registration of (serious) AE during and after treatment in (inter)national databases will help determine best practice in rTMS. Such registrations will help to identify potential relationships between (serious) AE and rTMS dependent factors (target area, frequency, intensity, number of sessions), psychiatric and somatic comorbidity, age, medication status. Future research in larger sample sizes is needed to tailor use of rTMS in LLD, and optimize efficacy while ensuring tolerance.

## CONFLICT OF INTERESTS

Odile A. van den Heuvel reports grants from Parkinson Vereniging, grants from Hersenstichting Nederland, grants from NWO‐ZonMw, other from Benecke, grants from National Institute of Mental Health, outside the submitted work. This has no influence on the submitted work. Remaining authors have no disclosure or financial or personal relationship with other people or organisations that could inappropriately influence the work.

## AUTHOR CONTRIBUTIONS

Geke M. Overvliet: Conceptualization, methodology, validation, formal analysis, investigation, resources, writing‐original draft and visualization. Rebecca A. C. Jansen: Methodology, validation, formal analysis, investigation, resources and writing‐original draft. Anton J. L. M. van Balkom: Writing‐review & editing. A. Dilene C. van Campen: Writing‐review & editing. Mardien L. Oudega: Writing‐review & editing. Ysbrand D. van der Werf: Writing‐review & editing. Eric van Exel: Writing‐review & editing. Odile A. van den Heuvel: Writing‐review & editing and supervision. Annemiek Dols: Conceptualization, methodology, validation, writing‐review & editing and supervision.

## DATA AVAILABILITY STATEMENT

Data is available on request.
